# Treatment of signs and symptoms of the common cold using EPs 7630 - results of a meta-analysis

**DOI:** 10.1016/j.heliyon.2019.e02904

**Published:** 2019-11-26

**Authors:** Andreas Schapowal, Gustav Dobos, Holger Cramer, Kian Chung Ong, Martin Adler, Andrea Zimmermann, Juliette Brandes-Schramm, Walter Lehmacher

**Affiliations:** a Allergy Clinic, Hochwangstraβe 3, 7302 Landquart, Switzerland; b Department of Internal and Integrative Medicine, Kliniken Essen-Mitte, Faculty of Medicine, University of Duisburg-Essen, Am Deimelsberg 34a, 45276 Essen, Germany; c KC Ong Chest & Medical Clinic, 3 Mount Elizabeth #12-03, Mount Elizabeth Medical Centre, Singapore 228510; d Institute of Integrative Medicine Siegen, University of Münster, Lärchenweg 27, 57078 Siegen, Germany; e Clinical Research, Dr. Willmar Schwabe GmbH & Co. KG, Willmar-Schwabe-Straβe 4, 76227 Karlsruhe, Germany; f Emeritus, University of Cologne, Institute of Medical Statistics, Informatics and Epidemiology, Kerpener Straβe 62, 50931 Cologne, Germany

**Keywords:** Respiratory system, Infectious disease, Pharmacology, Evidence-based medicine, Clinical research, Common cold, Efficacy, EPs 7630, Meta-analysis, *Pelargonium sidoides*, Safety

## Abstract

The efficacy of *Pelargonium sidoides* preparation EPs 7630 in the common cold (CC) was assessed by performing meta-analyses of randomized, double-blind, placebo-controlled trials. Mean differences (MD) and risk ratios (RR) with their 95% confidence intervals (CI) were computed. Five trials with a total of 833 patients were included. All trials had a treatment period of ten days with visits at days 3, 5, and 10 after baseline and used a ten-symptom Cold Intensity Score (CIS) as the primary outcome. Significant differences favoring EPs 7630 were observed for total CIS reduction (day 5: MD = -2·30; 95%CI = -4·12,-0·49; day 10: MD = -1·16; 95%CI = -2·22,-0·10), proportion of patients with substantial improvement (day 5: RR = 1·73; day 10: RR = 1·06) and complete remission (day 5: RR = 2·52; day 10: RR = 2·13). Subjects treated with EPs 7630 missed fewer days at work, used less paracetamol and had an improved sleep quality. No serious adverse reactions to EPs 7630 were reported. The results support the efficacy of EPs 7630 in adults with CC.

## 1 Introduction

The common cold (CC) is a highly prevalent, acute respiratory tract infection (RTI) of viral origin. It is one of the most common diseases occurring among all age groups. It is estimated that adults may experience two to five, and children may suffer from seven to ten colds per year (Eccles, 2005). Symptoms of CC are mainly related to the infected mucosa and affect the nose, sinuses, pharynx, larynx, and other large airways; they include nasal congestion and drainage, sneezing, coughing, sore throat, general malaise, and fever. Cold symptoms may appear as early as 10 h after infection and typically reach their maximum intensity at around three days after onset. Coughing in particular may still persist after three weeks (Heikkinen and Järvinen, 2003; Lorber, 1996).

The management of trivial RTIs such as CC is complicated by confusing terminology that has arisen to define their anatomic locations, while ignoring their usually diffuse nature (Manoharan and Winter, 2010). CC is a non-specific RTI whose characteristic symptoms partly overlap with other conditions such as acute bronchitis, allergic rhinitis, tonsillopharyngitis, rhinosinusitis, otitis media, and influenza. It is therefore not surprising that clinical trials in different acute respiratory infections often use similar diagnostic criteria. A clinical practitioner may consider an exact differential diagnosis to be of secondary importance as long as it does not imply a different type of treatment (e. g., in case of a bacterial infection or allergic rhinitis). However, despite the variability and overlap of symptoms, CC is considered to be a diagnostic entity in its own right.

Although CC is the most frequently encountered disease in primary care (Mossad, 1998), only a minority of patients with acute viral RTIs visit a physician (Fendrick et al., 2003). In Western countries CC is often treated through self-medication (Laven et al., 2014; Satoh et al., 2014), if at all. Nevertheless, symptoms of CC may interfere significantly with essential activities of daily living and may thus cause declines in function and productivity (Bramley et al., 2002; Smith et al., 2000). Consequently, the costs related to CC, e. g., through decreased productivity and time lost from work or school (indirect costs), visits to health-care providers, and the amount of drugs used (direct medical costs), are enormous (Bertino, 2002; Birnbaum et al., 2002; Fendrick et al., 2003). Treatment is therefore justified and motivated by a reduction of symptom burden and costs as well as by the prevention of more serious complications such as otitis media and pneumonia, as well as acute exacerbations of asthma or chronic obstructive pulmonary disease (Nahas and Balla, 2011).

While several drugs with in vitro activity against human rhinovirus, the leading cause of CC (Mäkelä et al., 1998), are currently under investigation (Bernard et al., 2014; MacLeod et al., 2013; Mello et al., 2014), there are yet no licensed effective antivirals for this condition, and therefore treatment aiming at symptom relief, a shortening of the illness duration, and a reduction of the risk of complications as well as of the infectivity to others remains the standard recommendation (Arroll, 2011; Picon et al., 2013). Symptomatic treatments of CC, including antihistamines, decongestants, non-steroidal anti-inflammatory drugs, paracetamol, and phyto-pharmaceutical products, have been extensively reviewed (Allan and Arroll, 2014; Arroll, 2011; Hemilä and Chalker, 2013; Kim et al., 2009; Mossad, 1998; Nahas and Balla, 2011; Science et al., 2012; Simasek and Blandino, 2007), albeit without providing clear, universally accepted therapeutic recommendations. Although the use of antibiotics is explicitly discouraged due to the predominantly viral etiology of the disease and the risk of adverse effects and resistances (Mäkelä et al., 1998), antibiotics over-prescription is still very common (Dekker et al., 2015; Gulliford et al., 2014), and further efforts are required to reduce inappropriate antibiotic use for the sake of containing costs and limiting the spread of antibiotic resistance.

Most reviews devoted to phytotherapy of CC assessed the effect of *Echinacea.* However, the results are difficult to interpret as *Echinacea* is not a single product, but the products used in clinical trials were based on different species and parts of the plants and used different methods of extraction. Moreover, different outcome measures and clinical scoring systems were used to assess treatment efficacy. While one review considered each identified trial individually (Nahas and Balla, 2011), the authors of another review performed a formal meta-analysis although the trials used different products based on different species and parts of the plants (Shah et al., 2007). This procedure was questioned by the authors of a recently updated Cochrane review on *Echinacea* (Karsch-Völk et al., 2014), who again refrained from pooling the results of trials investigating the efficacy of *Echinacea* products in the treatment of CC and argued that meta-analysis may only lead to meaningful results if all trials investigate the same treatment for the same purpose. Moreover, since some of the trials indicated a moderate beneficial effect of the investigated *Echinacea* products on CC duration and/or symptom intensity whereas others did not, it is not surprising that the efficacy conclusions drawn by the reviewers were mixed (Karsch-Völk et al., 2014; Nahas and Balla, 2011; Shah et al., 2007).

EPs 7630‡ is a herbal drug preparation from the roots of *Pelargonium sidoides* (drug – extract ratio: 1 : 8–10), extraction solvent: ethanol 11% (w/w), with antiviral and antibacterial activity as well as notable immune-modulatory capabilities (Moyo and Van Staden, 2014). The medicinal product is used both in adults and in children from the age of one year for the treatment of RTIs in several countries in Europe, Asia, Australia, Central and South America, and is available in three pharmaceutical forms, i. e. solution, film-coated tablets and syrup. In adults, the recommended daily dose is 30 drops of liquid solution or one 20 mg tablet thrice daily.

It is of interest that EPs 7630 is a single, well characterized phytopharmaceutical product so that an aggregation of information from several trials in the therapeutic indication of CC appears to be justified as shown in previous reviews for indications other than CC: for instance, in 2008 a first systematic review and meta-analysis suggested that EPs 7630 is effective for patients with acute bronchitis (Agbabiaka et al., 2008). A Cochrane review (Timmer et al., 2013) assessed the efficacy of EPs 7630 in various acute RTIs. Moreover, a meta-analysis published by Matthys et al. (2016) reviewed and supported the efficacy and safety of EPs 7630 in children, adolescents and adult patients with acute bronchitis, acute rhinosinusitis and acute tonsillopharyngitis. In 2018, a review showed that EPs 7630 is effective and safe for pediatric patients with acute bronchitis, acute tonsillopharyngitis and acute RTIs in the context of chronic preconditions (Careddu and Pettenazzo, 2018). The most recent meta-analysis of EPs 7630 in RTIs was published in 2019 and involved children suffering from acute tonsillopharyngitis or acute bronchitis. In these patients, EPs 7630 alleviated symptoms, accelerated recovery and reduced the concomitant use of paracetamol (Seifert et al., 2019).

In the current work, we present and discuss important challenges arising during the investigation of the efficacy of EPs 7630 in the treatment of CC. In a difficult-to-investigate indication like CC, where the effect sizes observed in clinical trials are variable, meta-analysis may be helpful for achieving a higher statistical power and obtaining more robust point estimates than from clinical trials reviewed individually. Moreover, exploratory, post-hoc meta-analyses may also investigate outcome measures of interest that need not necessarily have been pre-specified as such in the original protocols of the trials entered into the analyses (e. g., Haidich, 2010).

For EPs 7630, the therapeutic evidence for adults having CC with acute rhinosinusitis as an overlapping symptom has already been included in a European guideline, and the recommendation for viral and post-viral acute rhinosinusitis is directly based on category I evidence (Fokkens et al., 2012). The updated Cochrane review on EPs 7630 prepared by Timmer et al. (2013) assesses the efficacy of the herbal medicinal product in acute respiratory infections (acute bronchitis, sinusitis, CC, sore throat). For CC, the authors concluded that the herbal drug may be effective in providing symptom alleviation, but the efficacy of EPs 7630 in CC was difficult to evaluate because data from only a single randomized, placebo-controlled trial (Lizogub et al., 2007) had been published when the review was performed.

In order to present the complete clinical evidence with respect to efficacy and tolerability of EPs 7630 in CC, we performed the first review and meta-analysis of double-blind, randomized, placebo-controlled, therapeutic clinical trials with EPs 7630 in the indication of CC completed by October 2014, also including hitherto unpublished data.

## 2 Methods

### 2.1 Search strategy and selection criteria

Double-blind, randomized, placebo-controlled, therapeutic clinical trials with EPs 7630 in the indication of CC were eligible. Trials were identified from clinical trial registries (ISRCTN; Clintrials.gov), medical literature (MEDLINE), using the search term ‘EPs 7630’, and from the European Medicines Agency's assessment report on *Pelargonium sidoides* which was based on both published and otherwise unpublished data (Committee on Herbal Medicinal Products (HMPC), 2012). Moreover, the manufacturer of EPs 7630 was contacted for clinical trials meeting our eligibility criteria to identify any unpublished material.

### 2.2 Outcome measures

Severity of disease was assessed in all clinical trials using a disease specific, observer rated Cold Intensity Score (CIS) that included the symptoms nasal drainage, sore throat, nasal congestion, sneezing, scratchy throat, hoarseness, cough, headache, muscle aches, and fever. Each symptom was rated on a 5-point verbal rating scale ranging from 0 (‘not present’) to 4 (‘very severe’). A total score was computed by adding up the scores of the 10 individual symptoms (theoretical range: 0–40 points).

We assessed total CIS as well as individual symptom change versus baseline and performed responder analyses based on complete remission (defined as a total CIS of 0) as well as on substantial improvement (defined as an item score ≤1 for each of the symptoms included in the CIS). Other efficacy outcome measures of interest included in our analyses were the number of days until the onset of a meaningful treatment effect (according to the assessment of the patients), the number of days off work due to CC, paracetamol use, as well as the 1-item Integrative Medicine Outcomes Scale (IMOS) (Steinsbekk et al., 1999). Sleep quality was investigated using a 7-item subscale of the validated SF-A sleep questionnaire that describes the quality of sleep during the previous night (Görtelmeyer, 1986).

Tolerability was assessed based on adverse events.

### 2.3 Statistics

Meta-analyses for continuous variables were based on the mean value difference between the treatment groups and the associated 95% confidence intervals in their original scale. The same procedure was used for discrete, ordinal outcomes for the sake of illustration. Meta-analyses of binary outcomes were based on risk ratios and their 95% confidence intervals. Heterogeneity between the trials was assessed using the I^2^ statistic. Random effects models were computed in case of I^2^ > 5%, and fixed effect models were used otherwise. Review Manager (RevMan) Version 5·2 software was used for all meta-analyses (Anonymous, 2012). Treatment differences were considered descriptively significant if the 95% confidence interval of point estimate did not include the value of 0 for differences between means or of 1 for risk ratios. Missing data at days 5 and 10 were estimated using a last observation carried forward approach which was considered conservative in a self-limiting disease like CC (baseline data were not carried forward).

Tolerability was analyzed based on pooled data from all trials using risk differences and 95% confidence intervals. Adverse drug reactions (ADRs) listed in the company's reference safety information of the marketed product as potential unwanted effects were assigned to system groups of gastrointestinal complaints, hypersensitivity reactions, nasal bleeding, gingival bleeding, and liver associated events, which reflect adverse drug reactions that may occur seldom (i. e., in 1–10 patients out of 10,000 exposed) or occasionally (i. e., in 1–10 patients out of 1,000 exposed) during treatment with EPs 7630. Events were considered to be potentially related if a causal relationship to the blinded investigational treatment could not be excluded. 95% confidence intervals for the observed event rates within the treatment groups were determined using Wilson's score method (Newcombe, 1998b). Confidence intervals for event rate differences were computed according to Wilson's score method for the single proportion without continuity correction (Newcombe, 1998a).

The analyses were based on the Full Analysis Sets (FAS; for efficacy) and on the Safety Analysis Sets (SAF) of the original trials. For all trials except one both sets were identical (see Table 1). In another clinical trial, patients who received 3 x 30 and 3 x 60 drops/day were analyzed separately for efficacy outcomes.

**Table 1:** Characteristics of trials included in the meta-analysis.

Clinical trial	Country	Clinical part completed (year)		Formulation	Daily dose
A	Ukraine	2004	CIS: Sum of total score differences day 3 vs. baseline and day 5 vs. baseline	Liquid solution	3 x 30 drops, 3 x 60 drops
B	Ukraine	2004	CIS: Sum of total score differences day 3 vs. baseline and day 5 vs. baseline	Tablets	3 x 40 mg
C	Germany	2008	Total CIS: AUC, baseline through day 5	Liquid solution	3 x 30 drops
D	Bulgaria	2009	Total CIS: AUC, baseline through day 5	Liquid solution	3 x 30 drops
E	Singapore/Malaysia	2009	Total CIS: AUC, baseline through day 5	Tablets	3 x 20 mg

### 2.4 Role of the funding source

The clinical trials eligible for our meta-analyses were sponsored by Dr. Willmar Schwabe GmbH & Co. KG, Karlsruhe, Germany, manufacturer of EPs 7630, who also provided the subject data and performed the analyses according to the pre-specifications conceived by the authors.

## 3 Results

### 3.1 Eligible trials

We identified five trials (A through E) conducted between 2003 and 2009, which were performed according to similar protocols whose main characteristics are shown in Table 1. Trials A (Lizogub et al., 2007; Riley et al., 2018) and B (Riley et al., 2019) are published. Safety data from trials C and D were included in a safety review (Matthys et al., 2013) and trial E has not previously been published. As confirmed by the manufacturer, this review thus includes evidence from all randomized, placebo-controlled clinical trials performed with the herbal medicinal product in the indication common cold. Trials A and B were conducted in Ukraine, trial C in Germany, trial D in Bulgaria, and trial E in Singapore and Malaysia. Between 2 and 11 active trial sites participated in each clinical trial.

The participants of the trials were 833 male or female, adult out-patients (EPs 7630 417; placebo 416) who suffered from CC. Patient characteristics are presented in Table 2. The diagnosis was assured either (a) by the presence of nasal drainage and sore throat as primary CC symptoms and at least one (trials A + B) or two (trials C-E) of the secondary symptoms nasal congestion, sneezing, scratchy throat, hoarseness, cough, headache, muscle aches, and fever, or (b) by the presence of one of the primary symptoms and at least three of the secondary symptoms. Moreover, patients had to be suffering from CC symptoms for a maximum of 72 h prior to inclusion in the trial (trials C-E; trials A + B: 24–48 h). In all trials, patients were excluded if they had been taking medicines that could interfere with the interpretation of the results, including cold medications, within at least 4 days prior to enrolment. Furthermore, concomitant cold medications other than the trial medication and paracetamol were prohibited. Patients with co-morbidities in the respiratory system that could impair the interpretation of the results or patients with other relevant diseases were excluded from participation.

**Table 2:** Patient characteristics (percent or mean and standard deviation).

Clinical trial		Treatment	Number of patients	Sex:% female	Age (years)	Body weight (kg)	Body mass index (kg/m^2^)
A	3 x 30 drops/day	EPs 7630	52	69·2%	34·5 (10·60)	71·3 (15·12)	24·2 (4·01)
		Placebo	51	68·6%	37·4 (10·52)	70·7 (12·63)	24·3 (3·46)
	3 x 60 drops/day	EPs 7630	52	73·1%	36·8 (9·91)	70·6 (11·36)	24·8 (3·71)
		Placebo	52	76·9%	33·8 (10·84)	68·4 (12·96)	23·9 (3·83)
B		EPs 7630	53	75·5%	35·0 (10·86)	71·7 (12·88)	25·1 (3·86)
		Placebo	52	78·8%	37·7 (10·48)	73·6 (15·55)	25·7 (4·52)
C		EPs 7630	99	66·7%	37·1 (13·58)	73·0 (16·19)	24·8 (4·27)
		Placebo	101	65·3%	37·1 (12·46)	74·7 (15·91)	25·0 (4·42)
D		EPs 7630	101	63·4%	44·8 (14·10)	73·0 (18·16)	25·4 (4·84)
		Placebo	100	70·0%	46·2 (14·09)	69·9 (14·14)	24·7 (3·99)
E		EPs 7630	59^a^	44·1%	32·6 (11·02)	65·7 (17·53)	23·9 (5·94)
		Placebo	60	48·3%	33·3 (10·64)	64·0 (16·17)	23·2 (5·45)

a Applies to efficacy; safety: n = 60.

In all trials, randomized patients were treated for a scheduled period of ten days. Assessments were performed at baseline (day 1) as well as at days 3, 5, and 10. Trial E was stopped prematurely due to an outbreak of a H1N1 virus pandemia in 2009 in the countries where it was performed, since potential subjects were reluctant to accept a 50% chance of receiving placebo.

All trials were planned, executed and analyzed under consideration of the principles of Good Clinical Practice and the Declaration of Helsinki. The clinical trial protocols and other required documents were approved by the competent independent ethics committees and regulatory authorities. All trial participants provided informed consent.

### 3.2 Analysis of overall effect

**Fig. 1:**
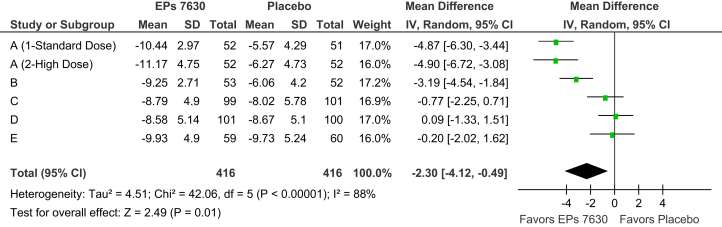
Meta-analysis of change of total CIS between baseline and treatment day 5 (FAS).

**Fig. 2:**
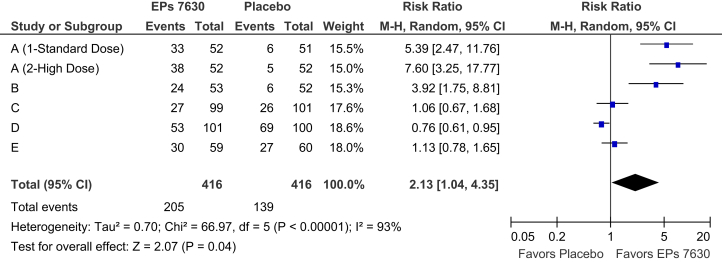
Meta-analysis of complete remission of all symptoms contained in the CIS until day 10 (FAS).

At baseline, the average total CIS in the five trials ranged between 14·9 and 17·8 points for the herbal drug and between 14·7 and 17·1 points for placebo, with higher baseline symptom burdens in trials A and B. This resulted in considerable heterogeneity between the mean values for total CIS change observed in the trials under investigation. As an example, Fig. 1 presents the average treatment group differences and their 95% confidence intervals for absolute total CIS change between baseline and treatment day 5. Whereas trials A and B show large treatment effects and significant superiority of EPs 7630 over placebo, moderate effects favoring the herbal extract were observed in trials C and E while trial D showed marginal differences between the treatment groups. Similar heterogeneity between the results of the five trials was also observed for complete remission of all CC symptoms at or before day 10 (Fig. 2) as well as for other efficacy outcome measures. A comparison between the results of the clinical trials also indicates that lower than average treatment group differences were associated with a higher than average response in the placebo group.

A review of the pooled meta-analysis results reveals a monotonic decrease of the total CIS in both treatment groups between baseline and the final visit at day 10. For total score change versus baseline (Table 3), superiority of EPs 7630 over placebo could already be observed at day 3 (p = 0·05), peaked at day 5 (Fig. 1; p = 0·01) and was still significant at day 10 (p = 0·03). Table 3 also shows that only a small number of patients were symptom free already after three or five days of treatment whereas 205/416 patients (49·3%) in the EPs 7630 group and 139/416 patients in the placebo group (33·4%) were in complete remission at day 10 (Fig. 2; p = 0·04). Advantages for the herbal extract were also observed for substantial improvement, notably at day 5 when improvement rates of 43·8% and of 31·5% were determined for EPs 7630 and placebo, respectively, corresponding to a pooled meta-analysis risk ratio of 1·73 (p = 0·02).

**Table 3:** Meta-analysis results for the Cold Intensity Score: total score change, complete remission, and substantial improvement (FAS).

	Visit	Responders		Point estimate and 95% CI
		EPs 7630 (n = 416)	Placebo (n = 416)	
Total score: change versus baseline (mean score difference) a	Day 3			-0·93 [-1·87; 0·02]
	Day 5			-2·30 [-4·12; -0·49]
	Day 10			-1·16 [-2·22; -0·10]
Complete remission (risk ratio) b	Day 3	2	1	1·67 [0·22; 12·56]
	Day 5	19	7	2·52 [1·13; 5·64]
	Day 10	205	139	2·13 [1·04; 4·35]
Substantial improvement (risk ratio) b	Day 3	34	39	0·89 [0·58; 1·35]
	Day 5	182	131	1·73 [1·08; 2·08]
	Day 10	368	343	1.06 [1·00; 1·13]

a Point estimates <0 favor EPs 7630.

b Point estimates >1 favor EPs 7630.

**Fig. 3:**
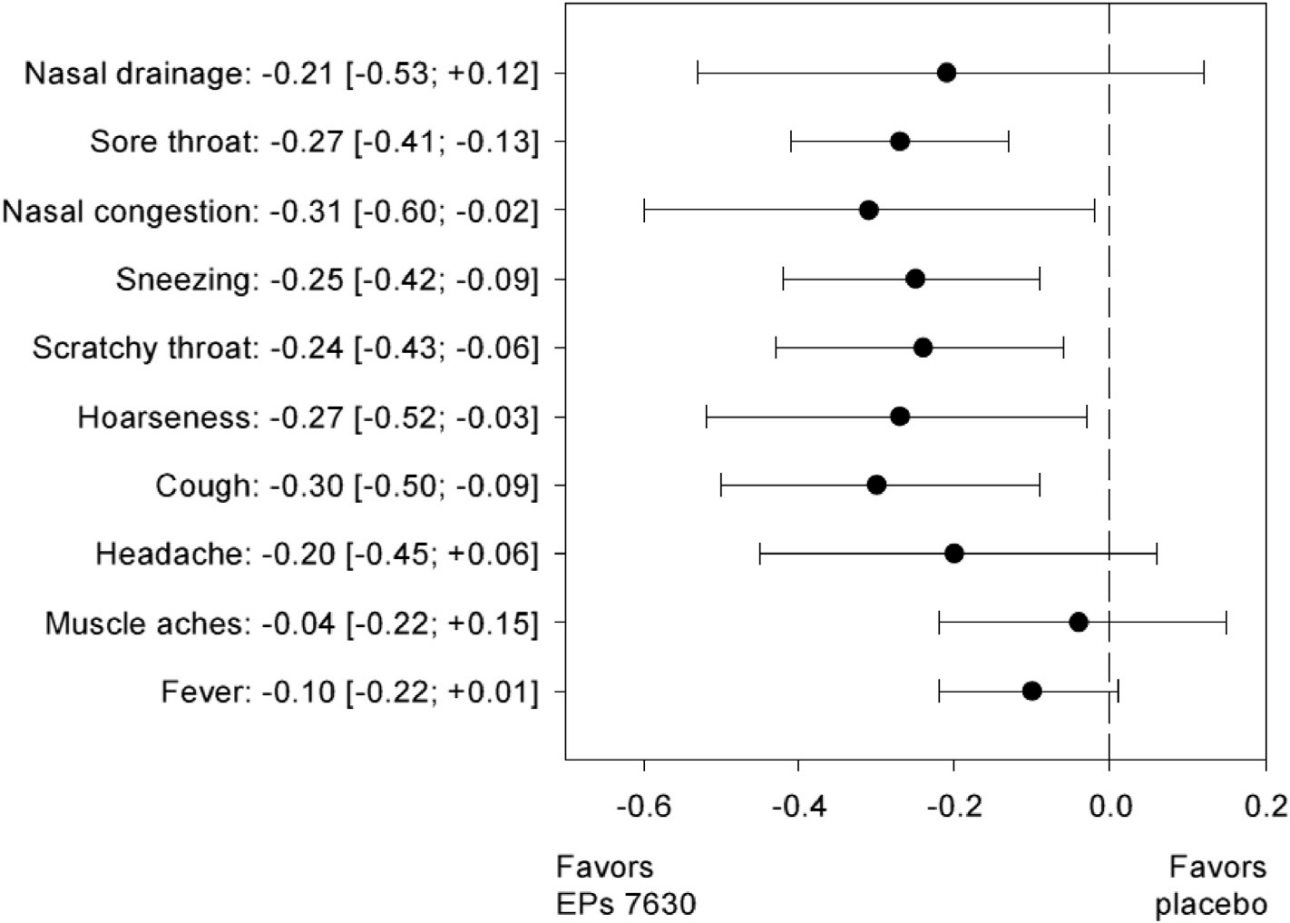
Change of individual CIS symptoms between baseline and treatment day 5 – overall meta-analysis results (mean value difference; FAS).

Fig. 3 presents the overall meta-analysis results for the treatment group comparison of the individual CIS symptoms for change between baseline and day 5. EPs 7630 was more efficacious than placebo in reducing all symptoms included in the CIS, with significant differences for sore throat, nasal congestion, sneezing, scratchy throat, hoarseness, and cough.

Meta-analysis main results for additional efficacy outcome measures are presented in Table 4. Days missed at work due to CC showed a large between-trial variability, with averages ranging between 0·6 and 5·9 days in the EPs 7630 group and between 1·0 and 6·7 days in the placebo group, from which a pooled mean value difference of 0·74 days favoring the herbal preparation was determined (Table 4; p = 0·01). In both treatment groups, the lowest average number of days off work was observed in trial A, whereas the highest numbers were observed in trials D and E (see Table 1).

**Table 4:** Meta-analysis results for other efficacy related outcome measures (FAS).

Outcome measure	N		Mean value difference and 95% CI
	EPs 7630	Placebo	
Days off work^a^	412	409	-0·74 [-1·33; -0·15]
Paracetamol consumption (mg)^a^,^c^	416	416	-79·0 [-152·4; -5·5]
Days until the onset of a treatment effect^a^	393	395	-1·12 [-2·14; -0·10]
IMOS – investigator rating, day 5^a^	416	416	-0·39 [-0·72; -0·06]
Sleep quality – sum of item scores, day 5^b^	405	401	1·63 [0·45; 2·81]

a Point estimates <0 favor EPs 7630.

b Point estimates >0 favor EPs 7630.

c Patients who did not use paracetamol were included in the calculation with a value of 0.

**Fig. 4:**
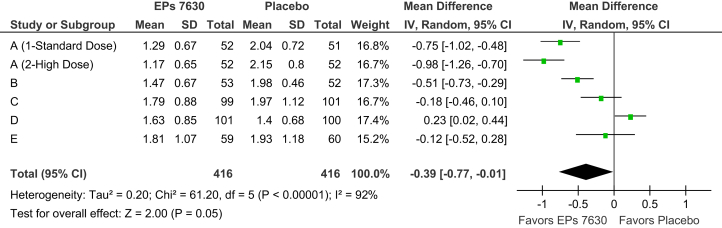
Meta-analysis of IMOS investigator rating, day 10 (FAS).

Among the five trials, the average time until the onset of a treatment effect ranged between 5·2 and 6·9 days for EPs 7630 and between 5·0 and 8·4 days for placebo. In the meta-analysis, the patients treated with the herbal preparation showed a more rapid onset of the treatment effect by a pooled difference to placebo of 1·1 days (p = 0·03). 54/416 patients in the EPs 7630 group (13·0%) and 77/416 patients in the placebo group (18·5%) used paracetamol at least once. The amount of paracetamol used was significantly lower in patients treated with the herbal preparation (p = 0·04). According to the IMOS, the patients treated with EPs 7630 had a more favorable overall outcome at days 5 (p = 0·02) and 10 (Fig. 4; p = 0·05). Moreover, compared to placebo, their quality of sleep was significantly better (days 5 and 10: p < 0·01).

### 3.3 Tolerability

Among the system groups mentioned in the company's reference safety information of the marketed product containing EPs 7630 (Table 5), increases of event rates in patients treated with the herbal drug by more than 1% compared to placebo were observed for gastrointestinal complaints, epistaxis, and for all events (including those from system groups not shown in the table). For all other system groups investigated, the incidence rates of adverse events under EPs 7630 were similar to those in patients treated with placebo, with point estimates for the risk difference not exceeding +0·5%. Our meta-analysis of all five trials, including events with any causal relationship to EPs 7630, revealed a pooled risk ratio of 1·51 (95% CI: [1·07; 2·13]; p = 0·02) favoring placebo. No serious adverse reactions to EPs 7630 were reported in any of the trials.

**Table 5:** Incidence of adverse events based on pooled data – number (%) of patients and 95% confidence intervals.

System group	Type	EPs 7630 (n = 417)	Placebo (n = 416)	Risk difference
Gastrointestinal complaints	All events	26 (6·24%) [4·29%; 8·98%]	19 (4·57%) [2·94%; 7·02%]	1·67% [-1·46%; 4·86%]
	Potentially related events	25 (6·00%) [4·09%; 8·70%]	19 (4·57%) [2·94%; 7·02%]	1·43% [-1·68%; 4·58%]
Hypersensitivity reactions	All events	5 (1·20%) [0·51%; 2·78%]	3 (0·72%) [0·25%; 2·10%]	0·48% [-1·06%; 2·12%]
	Potentially related events	3 (0·72%) [0·24%; 2·09%]	2 (0·48%) [0·13%; 1·74%]	0·24% [-1·10%; 1·66%]
Epistaxis	All events	11 (2·64%) [1·48%; 4·66%]	6 (1·44%) [0·66%; 3·11%]	1·20% [-0·84%; 3·36%]
	Potentially related events	9 (2·16%) [1·14%; 4·05%]	4 (0·96%) [0·37%; 2·45%]	1·20% [-0·60%; 3·18%]
Gingival bleeding	All events	–	–	–
	Potentially related events	–	–	–
Liver associated events	All events	1 (0·24%) [0·04%; 1·35%]	1 (0·24%) [0·04%; 1·35%]	-0·00% [-1·13%; 1·12%]
	Potentially related events	1 (0·24%) [0·04%; 1·35%]	0 (0·00%) [0·00%; 0·91%]	0·24% [-0·70%; 1·35%]
All system groups^a^	All events	66 (15·83%) [12·64%; 19·64%]	44 (10·58%) [7·97%; 13·90%]	5·25% [0·64%; 9·87%]
	All potentially related events	39 (9·35%) [6·92%; 12·53%]	25 (6·01%) [4·10%; 8·72%]	3·34% [-0·30%; 7·05%]

a Also includes events from system groups not shown in this table.

In trial A, higher AE rates were observed for the 3 x 60 drops/day dose as compared to the 3 x 30 drops/day dose. This applied to both EPs 7630 (15·4% vs. 7·7%) and placebo (5·8% vs. 3·9%).

## 4 Discussion

### 4.1 Clinical efficacy

Our results demonstrate that EPs 7630 is significantly superior to placebo in alleviating the symptoms of the CC. Should results be aggregated using meta-analysis methods when substantial heterogeneity exists, with I^2^ values of 88% and 93% for total score change at day 5 and complete remission at day 10, respectively? The I^2^ measure can be thought of as an indicator of the proportion of variance that reflects true differences in effect size between the trials in a meta-analysis (Higgins et al., 2003). As such, it reflects the extent by which the confidence intervals for the effect size point estimates overlap. Our interpretation that the observed differences favoring EPs 7630 in the pooled effect measures of our meta-analysis represent a true treatment effect rather than bias is supported by the fact that benefits of the herbal preparation were consistently observed in four of the five clinical trials included in the review although the treatment effect sizes in two of these trials were admittedly small to moderate (Ioannidis, 2008). Heterogeneity observed between the clinical trials was therefore mainly attributable to disagreement in the magnitude, not in the direction of the EPs 7630 treatment effect. Moreover, when investigating a drug with no pharmacological effects one would rather expect to find only chance differences to placebo, some of them favoring the investigational drug and some placebo treatment. This was clearly not the case for the clinical trials presented in this meta-analysis. Moreover, bias caused by selective reporting can also be excluded since all trials investigating EPs 7630 in the indication of CC and completed until the compilation of our meta-analysis were included in the review.

In clinical trials, overlapping symptoms between indications such as acute bronchitis and CC almost inevitably lead to overlapping inclusion criteria and/or efficacy outcome measures. It is therefore essential to assure that the participants of a trial actually suffer from the particular diagnostic entity under investigation. It is also noteworthy in this context that to date, the CIS, which is based on a validated score initially developed by Jackson and coworkers (Jackson et al., 1958; Mossad et al., 1996; Prasad et al., 2000), is one of the few observer rated instruments for assessing the symptoms of CC, and moreover, symptoms severity assessments, obtained predominantly through patient diaries, are necessarily subjective and may thus introduce bias (Barrett et al., 2002; Mossad et al., 1996). Both symptom overlap and the lack of formally validated symptom severity scales may contribute to heterogeneity between the results of different trials.

To explain the heterogeneity between the clinical trials, different statistical models including factors possibly responsible for the heterogeneity were investigated.

### 4.2 Heterogeneity between clinical trial inclusion criteria

From a clinical point of view, comparing the inclusion criteria of the clinical trials, the main difference between trials A, B on one side and trials C, D, E on the other side is the acceptable delay between the onset of CC symptoms and the inclusion in the clinical trial. In trials A and B, the delay was between 24 and 48 h compared to between 24 and 72 h in the other three trials. The higher initial symptom burden in trials A and B may explain some of the high heterogeneity between trial results.

In other common cold trials with results demonstrating superiority over placebo using other products, treatment was initiated promptly when patients had a first subjective feeling of CC because the incubation period varies but is just under two days for rhinovirus (Allan and Arroll, 2014; Hoheisel et al., 1997; Mossad et al., 1996; Prasad et al., 2000; Schulten et al., 2001).

### 4.3 Heterogeneity between clinical trial populations

Another source of heterogeneity may be cultural or socioeconomic differences between trial populations that are not related to the type or intensity of symptoms. As an example, days missed at work due to CC showed a large between-trial variability. In both treatment groups, the lowest average number of days off work was observed in trial A, whereas the highest numbers were observed in trials D and E. The results thus point to differences regarding the subjectively perceived disease burden and/or the inclination to take (or to provide) sick leave between the countries where the trials were performed.

### 4.4 Agreement between different outcome measures within clinical trials

The degree of concordance between the different outcome measures within an investigation may be an important indicator for the internal validity of the primary results.

The meta-analysis results based on the total CIS are supported by the fact that benefits of the herbal preparation were consistently observed for symptomatic improvement, general medical outcome, and other disease related measures. Our results indicate that compared to placebo, EPs 7630 alleviated the symptoms of CC and decreased the time until substantial improvement and complete remission, resulting in fewer days missed at work. Moreover, treatment with the herbal preparation reduced the need for paracetamol consumption and resulted in improved sleep quality. Based upon a much broader database, this analysis therefore confirms and extends the conclusions drawn from previous research according to which EPs 7630 may offer symptom relief in CC (Timmer et al., 2013).

### 4.5 Efficacy in a short-term disease

CC is characterized by a comparatively short course of disease when no complications arise. In a therapeutic clinical trial, this implies a narrow window for the initiation of treatment after the appearance of the first symptoms, as well as a clinically meaningful timing of assessments to assure comparability of results. Since most symptoms of CC are known to subside within about 10 days untreated (Heikkinen and Järvinen, 2003; Lorber, 1996), the pharmacological effect of an intervention will likely be masked by the natural course when the period of observation extends beyond day 10.

In the analysis performed for EPs 7630, superiority of the herbal extract over placebo was already detectable at the first post-baseline visit (day 3), and the effect was most pronounced at the second post-baseline visit (day 5; Table 3). Although the decrease of CC associated symptoms was likely increasingly confounded with the natural course of the disease as treatment progressed, the differences between EPs 7630 and placebo regarding total CIS change, substantial improvement, and complete remission were still significant at day 10 after baseline.

### 4.6 Tolerability

The EPs 7630 meta-analysis of safety data points to a moderate increase of the AE rate in general and of gastrointestinal complaints and epistaxis. This is consistent with the results of an extensive safety review performed by Matthys and colleagues based on data from 29 clinical trials and non-interventional studies, with a total of more than 8,000 participants exposed to EPs 7630, in which the authors found slight increases of the risk of gastrointestinal disorders and epistaxis in patients receiving the herbal extract (Matthys et al., 2013). However, compared to placebo, only a slight overall increase in the frequency of adverse events in the EPs 7630 groups was observed. AEs potentially related to the trial treatment (EPs 7630 or placebo) were comparable between groups, and no serious adverse events occurred in any of the groups.

## 5 Conclusion

The common cold is a common disease that usually has an uncomplicated course. Nevertheless, and partly because of this, the efficacy of therapeutic interventions in CC are difficult to assess. Challenges faced by investigators include symptom overlap with other acute RTIs, as well as sociocultural and other differences between trial populations that influence the patients’ subjective perception of the disease as well as of its consequences, such as whether or not they consult a physician or remain home from work. Together with a comparatively narrow therapeutic window caused by the short natural course, these factors contribute to substantial heterogeneity between trial results that has been observed in reviews and meta-analyses for a variety of CC treatments.

In this regard, the clinical trials performed for *Pelargonium sidoides* extract EPs 7630 are no exception. Nevertheless, our meta-analysis supports the efficacy and safety of the drug in adults with CC. Treatment with the herbal preparation was associated with symptom alleviation and more rapid remission and may thus not only reduce the burden on the individual patient, but also the burden on the healthcare system and the economic impact of this very common condition. More evidence would be helpful due to the heterogeneity of the trial results that made the treatment effect of EPs 7630 difficult to assess. The results also confirm that this herbal preparation is well-tolerated.

## Declarations

### Author contribution statement

All authors listed have significantly contributed to the development and the writing of this article.

### Funding statement

This research did not receive any specific grant from funding agencies in the public, commercial, or not-for-profit sectors.

### Competing interest statement

The authors declare the following conflict of interests: Andreas Schapowal, Gustav Dobos, Kian Chung Ong, Martin Adler, and Walter Lehmacher have received honoraria from Dr. Willmar Schwabe GmbH & Co KG, Karlsruhe, Germany; Andrea Zimmermann and Juliette Brandes-Schramm are employees of Dr. Willmar Schwabe GmbH & Co. KG, Karlsruhe, Germany. Holger Cramer declares no conflict of interest.

### Additional information

No additional information is available for this paper.

## Appendix A Supplementary data

icmje-coi-form_Dobos icmje-coi-form_Dobos

## Footnotes

‡ EPs® 7630 is the active ingredient of the product Umckaloabo® (ISO Arzneimittel, Ettlingen, Germany).
